# Inner-Frame Time Division Multiplexing Waveform Design of Integrated Sensing and Communication in 5G NR System

**DOI:** 10.3390/s23156855

**Published:** 2023-08-01

**Authors:** Jian Zheng, Ping Chu, Xiaoye Wang, Zhaocheng Yang

**Affiliations:** 1 College of Electronics and Information Engineering, Shenzhen University, Shenzhen 518060, China; 2110436094@email.szu.edu.cn (J.Z.); yangzhaocheng@szu.edu.cn (Z.Y.); 2 College of Computer Science and Engineering, Huizhou University, Huizhou 516007, China; wangxiaoye@hzu.edu.cn

**Keywords:** OFDM, 5G new radio (NR), integrated sensing and communication, waveform design

## Abstract

The design of an integrated sensing and communication (ISAC) waveform compatible with the 5G new radio (NR) system is crucial in enabling ISAC by utilizing the hardware of existing base stations (BSs). In this paper, we design an inner-frame time division multiplexed sensing waveform in the frame structure of 5G NR to achieve ISAC. The designed waveform is computed by the simulated annealing algorithm on an optimization cost function of a constrained combination of the peak-to-sidelobe ratio (PSLR) and the integrated sidelobe ratio (ISLR) of the velocity ambiguity function. Specifically, the constraints are the 5G communication protocol and 5G NR frame structure. In addition, we conducted corresponding signal detection and estimation methods to illustrate the performance of the sensing waveform. Both theoretical analysis and simulation experiments show that the designed waveform can effectively achieve target detection and parameter estimation under low sensing cost conditions.

## 1. Introduction

The development of electronic technology has spurred increased interest and research in radar and communication equipment. Radar and communication devices are similar in terms of hardware architecture, channel characteristics, and signal processing [1]. Integrated sensing and communication (ISAC) systems can greatly improve spectrum and energy efficiency and reduce hardware and signal costs [2].

With the advent of 5G communication, the bandwidth of communication systems is gradually widening, and the carrier frequency is becoming higher. This makes a common radio frequency and baseband software radio platform for ISAC possible [3]. The most straightforward way to achieve ISAC is to reuse communication infrastructure for sensing, with a low-cost and fast-deployment footprint [4,5]. The key problem in ISAC lies in how to design the ISAC waveforms.

The ISAC waveforms can be divided into two main categories: multiplexing waveforms and identical waveforms. The multiplexing waveform typically utilizes techniques such as time division multiplexing [6,7], frequency division multiplexing [8,9], space division multiplexing [10], code division multiplexing [11,12], or their combinations. The multiplexing waveform can avoid interference between radar and communication, making waveform designs and signal processing relatively independent and simple. However, it might prevent the sharing of resources. Identical waveforms can be classified into sensing-centric waveforms and communication-centric waveforms [3,13]. Sensing-centric waveforms are modified radar waveforms where the communication information is embedded [14,15,16,17]. However, the sensing-centric waveform keeps the pulse regime of the radar waveform, so the communication data rate of this waveform is low. The communication-centric waveform is usually based on the traditional communication waveform. The most widely used communication-centric waveform is the orthogonal frequency division multiplexing (OFDM)-integrated radar and communication waveform.

OFDM waveforms provide high communication data rates and the ability to efficiently deal with frequency selective fading. Hence, many researchers have investigated the OFDM-integrated radar and communications waveform [18,19,20,21,22,23,24,25,26,27]. In [18,19], the authors proposed a waveform design method that maximizes the weighted sum of the communications data information rate (DIR) and the conditional mutual information (MI) between the observed signal and the random target impulse response over the entire uncertainty class. Moreover, Ref. [20] proposed an adaptive waveform design method by maximizing the mutual information (MI), subject to the subcarrier power ratio, energy constraints, and communication channel capacity. Ref. [21] considered the MI between the frequency-dependent target response and the transmit OFDM waveforms. Ref. [22] proposed minimizing the Cramer–Rao bound (CRB) on the delay-Doppler estimation, subject to an integrated side-lobe level (ISL). Ref. [23] transformed the optimal waveform design problem by designing subcarrier coefficients for optimal power allocation and phase coding. The above methods add a coefficient before the subcarrier of the OFDM waveform and use different optimization criteria to achieve the optimal waveform design. In addition, [24] proposed maximizing the energy efficiency (EE) and sum rate (SR) of the ISAC system. However, radar and communications require the use of different transceiver antennas. In [25], the concept of shared and private subcarriers is proposed, where shared subcarriers are used to realize the communication function, and private subcarriers are used to assist sensing. When the number of private subcarriers is increased, the sensing performance improves, but the communication rate decreases. In [26], a sensing-integrated discrete Fourier transform spread OFDM system is proposed for THz ISAC. The authors of [27] proposed a joint radar–communication system with offset quadrature amplitude modulation-based orthogonal frequency division multiplexing (OQAM-OFDM). Although communication-centric waveforms can achieve a high data rate, the sensing performance suffers due to the random autocorrelation property and the high peak-to-average-power ratio of the transmitted waveform.

With the advent of the 5G era, 5G base stations (BSs) have widely being deployed around the world. The utilization of existing 5G BS hardware to achieve ISAC offers advantages such as low cost and rapid deployment. Therefore, many researchers are looking at how to utilize existing 5G BS hardware to achieve ISAC [28,29,30]. Ref. [28] optimized the ISAC waveform by filling the empty subcarriers of a 5G BS working in downlink mode and optimizing the transmission power of some of the communication subcarriers. However, the communication waveform is random in nature, and the number of empty subcarriers in the communication depends on the number of users served by the networks. The data transmitted by the users exhibit bursts and discontinuity. Ref. [29] proposed a novel ISAC scheme that constructs three adjacent BSs as a cooperative sensing system. This scheme stipulates that only one of the three adjacent BSs can be working in downlink mode at any time. However, this scheme would reduce the BS’s duration in downlink mode by one-third, significantly reducing the communication data rate. Therefore, reusing the hardware of existing, widely deployed 5G BSs to achieve ISAC, while minimizing the impact on the existing communication data rate and avoiding the impact of the random autocorrelation property of communication signals on sensing performance, poses a complex issue, which is addressed in our proposed waveform design method.

In this paper, we propose an inner-frame time division multiplexing waveform design method of ISAC in 5G NR systems, which achieves communication and sensing by reusing the hardware of existing 5G BSs, characterized by its low cost, rapid deployment, and minimal impact on the communication data rate. We transform the sensing waveform design problem under 5G communication constraints into an optimization problem by combining the advantages of time division multiplexed waveforms and communication-centric waveforms. We use the simulated annealing algorithm to solve the optimization problem and obtain the optimized sensing waveform. The designed waveform takes into account the constraints on the BS communication signal in 5G systems and is fully compatible with existing hardware devices for 5G. For the designed waveform, we also employ the corresponding signal detection and estimation methods to extract the target information. The results of the theoretical analysis and numerous simulation experiments show the effectiveness of the designed waveform.

The remainder of this paper is organized as follows. In Section 2, the signal model is introduced in the 5G NR system. In Section 3, the sensing waveform is designed and the corresponding signal processing methods are detailed. In Section 4, numerical examples are presented to illustrate the performance of the designed waveform. In Section 5, the conclusions are drawn.

Hereafter, ${\left(\cdot \right)}^{-1}$, ${\left(\cdot \right)}^{T}$, ${\left(\cdot \right)}^{*}$, and ${\left(\cdot \right)}^{H}$ stand for the inverse, transpose, conjugate, and conjugate transpose of the argument, respectively; diag(A) and tr(A) represent the vector consisting of the diagonal elements of matrix A and the trace of matrix A, respectively; $\left\lfloor \cdot \right\rfloor$ represents rounding down, and the mod(a,b) represents the remainder of *a* divided by *b*.

## 2. Signal Model in the 5G NR System

**Figure 1:**
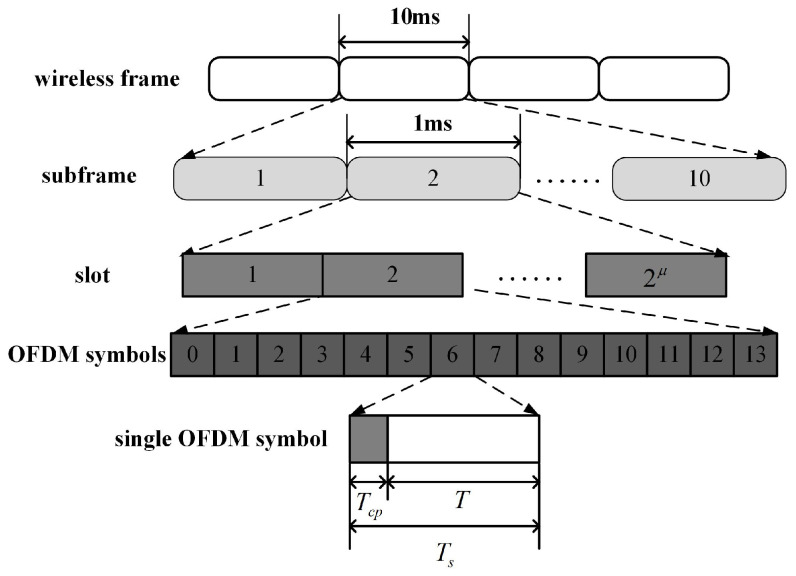
5G NR frame structure.

In the 5G NR system, the communication information is transmitted into the wireless frame, as shown in Figure 1, which contains 10 subframes. Each subframe consists of $2^{\mu }$ slots, where μ is one of the parameters in the 5G NR system. It can take values of 0, 1, 2, 3, or 4. For each slot, there are 14 completed OFDM symbols with lengths of $T_{s}$. Moreover, the completed OFDM symbol can be divided into two parts, the effective OFDM symbol with length *T* and the cyclic prefix (CP) with length $T_{cp}$. Specifically, $T_{s}=T_{cp}+T$, $T_{cp}=7\%\times T$, and T=1/Δf, where $\Delta f=2^{\mu }\times 15\mathrm{kHz}$ is the subcarrier interval. Hence, there are totally $N=10\times 14\times 2^{\mu }$ OFDM symbols within a wireless frame.

The baseband signal $s_{n}\left(t\right)$ of the *n*th OFDM symbol within a wireless frame can be expressed as

(1) $$s_{n}\left(t\right)=\sum_{m=0}^{N_{c}-1}\alpha_{m,n}e^{j2\pi m\Delta f(t-nT_{s})}\mathrm{rect}\left(\frac{t-nT_{s}}{T_{s}}\right),$$

where $\alpha_{m,n}$ is the communication information modulated on the *m*th subcarrier of the *n*th OFDM symbol; n=0, 1, …, N−1, $N_{c}$ is the number of subcarriers; and rect(t) is the rectangular function, defined as

(2) $$\mathrm{rect}\left(t\right)=\left\{\begin{array}{cc} 1, & 0\leq t\leq 1\\ 0, & \mathrm{otherwise}. \end{array}\right.$$

Then, the final transmitted OFDM waveform can be given by

(3) $${\bar{s}}_{n}\left(t\right)=s_{n}\left(t\right)e^{j2\pi f_{0}t},$$

where $f_{0}$ is the carrier frequency.

According to the 5G communication protocol [31], 5G communication supports the time division duplex (TDD). The TDD is divided into the uplink mode and downlink mode. When the 5G BS works in the uplink mode, it receives the OFDM communication signals sent by the users. When the BS works in the downlink mode, it transmits OFDM communication signals to the users. The BS switches continuously between uplink and downlink modes within one wireless frame. According to different communication demands, the uplink and downlink modes have different time ratios. We assume that the time ratio of the uplink and downlink modes is AS1, which is one of the time ratios specified in the 5G communication protocol, to provide clear constraints for the subsequent waveform design. Without loss of generality, the proposed method’s optimization approach is similar to other cases.

**Figure 2:**
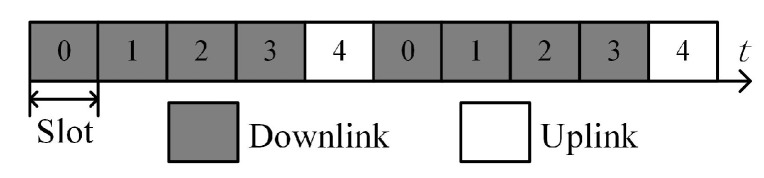
Downlink constraint.

Furthermore, there are common channels in the transmission of OFDM signals. The common channels are used to transmit various communication-related important parameters, including downlink control information and achieving synchronization between communication parties. The common channels include the synchronization signal/PBCH block (SSB), physical downlink control channel (PDCCH), and physical downlink shared channel (PDSCH), which are only distributed in the first half of a wireless frame with different modes. For the same reason as the AS1, we assume that the distribution of the common channel OFDM symbols is AS2, which is also derived from the 5G communication protocol, to provide clear constraints for the subsequent waveform design. AS1 and AS2 are as follows:

AS1: Downlink constraint. The first four of every five consecutive slots are fixed as the downlink, and the last slot is fixed as the uplink, as shown in Figure 2. Specifically, for the *n*th OFDM symbol, if $\mathrm{mod}\left(\left\lfloor n/14\right\rfloor ,5\right)\in \varepsilon_{1}$, it means that the *n*th OFDM symbol belongs to the downlink, otherwise it belongs to the uplink, where $\varepsilon_{1}=\left\{0,\;1,\;2,\;3\right\}$.

**Figure 3:**
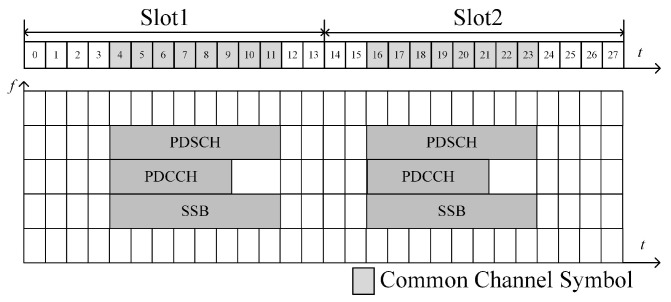
One of the common channel constraints.

AS2: Common channel constraint. The resource allocation for the common channel is considered to be the <120 kHz, 120 kHz> mode, as shown in Figure 3. Specifically, for the *n*th OFDM symbol in the first half of a wireless frame, if mod(n,(N/2))=0 and $\mathrm{mod}\left(n,28\right)\in \varepsilon_{2}$, it means that it is a common channel symbol, otherwise, it is not, where $\varepsilon_{2}=\left\{4,\;5,\;6,\;7,\;8,\;9,\;10,\;11,\;16,\;17,\;18,\;19,\;20,\;21,\;22,\;23\right\}$.

## 3. Proposed Sensing Waveform

In this section, we detail the proposed sensing waveform in terms of design strategy, optimization solution, and signal detection and estimation.

### 3.1. Proposed Waveform Design Strategy

Due to the shared hardware of the existing 5G BSs for ISAC, the 5G BSs are required to work in downlink mode. To minimize the impact on communication, waveform design methods need to avoid modifications to common channel symbols. In this paper, the above requirements are assumed to be AS1 and AS2, respectively. This will result in the sensing waveform being nonuniform. Additionally, to avoid the interference of communication signals on sensing and ensure minimal impacts on the communication, we focus on the inner-frame time division multiplexing of ISAC to exploit the millimeter-wave bands in 5G NR systems. Specifically, we extracted a few OFDM symbols for sensing within the wireless frame, and would like the sensing waveform to be as sparse as possible while ensuring acceptable sensing performance.

Regarding sensing performance, range resolution and Doppler resolution are two basic metrics. Since we use the inner-frame time division multiplexing, it is easy to obtain a high-range resolution by selecting a large bandwidth. But for the Doppler resolution, it relies on the slow-time sampling and observation time. The uniformly spaced intervals cannot be realized, which is a major challenge when designing sensing waveforms within the 5G NR frame structure. Therefore, this paper focuses on designing a sparse and nonuniform sensing waveform with acceptable detection and estimation performance.

Generally, the usual waveform design methods include detection probability-based, signal-to-noise ratio (SNR)-based, signal-to-interference-plus-noise ratio (SINR)-based, ambiguity function-based, and Kullback–Leibler scatter-based methods [32]. The ambiguity function is an important tool for evaluating the sensing parameters in the radar system. In order to remove the interference of modulation information on sensing, we set $\alpha_{m,n}=1$ in (1) and propose the sensing symbolic baseband model, given by

(4) $$s_{n}^{\prime }\left(t\right)=\sum_{m=0}^{N_{c}-1}e^{j2\pi m\Delta f(t-nT_{s})}\mathrm{rect}\left(\frac{t-nT_{s}}{T_{s}}\right).$$

Thus, the sparse and nonuniform sensing waveform can be expressed as

(5) $$S_{t}=Us_{t},$$

where U is an L×N sparse matrix whose *l*th row and *n*th column element $u_{l,n}\in \left\{0,\;1\right\}$, and $s_{t}={\left[s_{0}^{\prime }\left(t\right),\;s_{1}^{\prime }\left(t\right),\;\ldots ,\;s_{N-1}^{\prime }\left(t\right)\right]}^{T}$ is an N×1 vector. Specifically, each row of the U has one and only one $u_{l,n}=1$, and each column of the U has, at most, one $u_{l,n}=1$. For convenience, we define the column numbers of the non-zero elements in the *l*th row of U as $n_{l}$, for l=0, 1, …, L−1.

Hence, we compute the ambiguity function, expressed by

(6) $$\begin{array}{cc} \chi_{U}\left(\tau ,f_{d}\right)= & \int_{-\infty }^{\infty }S_{t-\tau }^{H}S_{t}e^{j2\pi f_{d}t}dt\\ = & \sum_{l=0}^{L-1}\sum_{m=0}^{N_{c}-1}\sum_{m^{\prime }=0}^{N_{c}-1}(T_{s}-\left|\tau |\right)e^{j\pi \left[\left(m-m^{\prime }\right)\Delta f+f_{d}\right]\left(T_{s}+\tau \right)}\\ & e^{j2\pi m^{\prime }\Delta f\tau }\mathrm{sinc}\{\pi \left[\left(m-m^{\prime }\right)\Delta f+f_{d}\right](T_{s}-\left|\tau |)\right\}e^{j2\pi f_{d}n_{l}T_{s}}. \end{array}$$

where τ is the round-trip propagation time, $\left|\tau \right|\leq T_{s}$, and $f_{d}$ is the Doppler frequency shift. Moreover, $\chi_{U}\left(\tau \right)$ represents the range ambiguity function when $f_{d}$ is fixed to 0 in (6), and Δf and $N_{c}$ are important parameters affecting the range estimation performance. Similarly, $\chi_{U}\left(f_{d}\right)$ represents the velocity ambiguity function when τ is fixed to 0 in (6), and U is an important parameter affecting the velocity estimation performance. Since we use inner-frame time division multiplexing, we can easily obtain an acceptable range estimation performance by picking appropriate parameters, but it is not easy to obtain an acceptable velocity estimation performance because of the nonuniform and sparse waveform. Therefore, the main problem of the sensing waveform design is how to design the proper parameter U to achieve acceptable velocity estimation performance. Two metrics can be used to characterize the velocity ambiguity function. They are the peak-to-sidelobe ratio (PSLR) and the integrated sidelobe ratio (ISLR), i.e., the ratio of the peak of the sidelobe to the peak of the main lobe and the ratio of the energy of the sidelobe to the main lobe, respectively. They are defined as

(7) $${\mathrm{PSLR}}_{U}=\frac{\max[\chi_{U}\left(f_{di}\right){|}_{f_{di}\notin \left[f_{a},f_{b}\right]}^{2}]}{\max[\chi_{U}\left(f_{di}\right){|}_{f_{di}\in \left[f_{a},f_{b}\right]}^{2}]},$$

(8) $${\mathrm{ISLR}}_{U}=\frac{\sum_{f_{di}}\chi_{U}\left(f_{di}\right){|}_{f_{di}\notin \left[f_{a},f_{b}\right]}^{2}}{\sum_{f_{di}}\chi_{U}\left(f_{di}\right){|}_{f_{di}\in \left[f_{a},f_{b}\right]}^{2}},$$

where $\left[f_{a},f_{b}\right]$ stands for the range of the main lobe.

Thus, under the constraints of AS1 and AS2, the proposed waveform design strategy employing inner-frame time division multiplexing can be described as the following optimization problem:

(9) $$\begin{array}{cc} & \;\;\;\;\;\;\;\;\;\;\;\;\;\;\;\;\;\min_{U}\;F\left(U\right)\\ & s.t.\left\{\begin{array}{c} \begin{array}{c} \mathrm{mod}\left(\left\lfloor n_{l}/14\right\rfloor ,5\right)\in \varepsilon_{1}\\ \mathrm{mod}\left(n_{l},28\right)\notin \varepsilon_{2},\;\forall \mathrm{mod}\left(n_{l},\left(N/2\right)\right)=0\\ \left\lfloor n_{i}/14\right\rfloor \neq \left\lfloor n_{j}/14\right\rfloor ,\;\forall i\neq j\\ L\leq K_{s}N \end{array} \end{array}\right. \end{array}$$

where F(U) is the cost function, defined as

(10) $$F\left(U\right)=\eta {\mathrm{PSLR}}_{U}+\left(1-\eta \right){\mathrm{ISLR}}_{U}.$$

Here, η is the scaling factor that determines the trade-off between the PSLR and ISLR. It should be noted that in (9), the first constraint is the downlink constraint in AS1, the second constraint is the common channel constraint in AS2, the third constraint consists of (at most) one OFDM sensing symbol in each slot, and the fourth constraint is the sparsity constraint. $K_{s}$ denotes the sparsity of the sensing waveform, defined as

(11) $$K_{s}=\frac{L_{s}}{N}\times 100\%,$$

where $L_{s}$ denotes the number of symbols used when the sparsity is $K_{s}$.

### 3.2. Waveform Solution Based on the Simulated Annealing Algorithm

For the optimization problem (9), the solution is NP-hard. However, we do not need to find the optimal solution. We only need to find a solution that satisfies the constraints and meets the requirements of the sensing scenario. Therefore, we can use a suboptimal solution as a substitute for the optimal one. Traditional solving algorithms include simulated annealing [33], genetic algorithms, ant colony algorithms, and so on. For simulated annealing, it is easy to consider each sensing OFDM symbol as a particle inside an object. As the temperature decreases, the particles tend to stabilize. Eventually, the internal energy reaches a minimum, resulting in an optimal solution. In theory, the other algorithms mentioned above are also feasible, but encoding the solving process of the optimization problem (9) into the corresponding physical processes of other algorithms is difficult. Therefore, we use the simulated annealing algorithm to solve the optimization problem (9). A summary of the simulated annealing algorithm is shown in Algorithm 1.

Step 1: Initialize the temperature of the system as $T_{temp}=T_{max}$, generate a U randomly, according to (9), and obtain the sensing waveform $S_{t}$ according to (5).

Step 2: Calculate the number of fluctuation symbols $N_{r}$ and the fluctuation range based on $T_{temp}$. Select $N_{r}$ non-zero elements in U to exchange with zero elements in the fluctuation range and judge whether (9) is satisfied. If not, repeat Step 2, and if satisfied, generate a new $U^{\prime }$. Then, obtain a new sensing waveform $S_{t}^{\prime }$ according to (5).

Step 3: Calculate the cost functions F(U), $F(U^{\prime })$ according to (6)–(10) and $\Delta F=F\left(U^{\prime }\right)-F\left(U\right)$.

Step 4: If ΔF≤0, the new solution $U=U^{\prime }$ and $S_{t}=S_{t}^{\prime }$ is obtained. If ΔF>0, the new solution is received when $e^{-\Delta F/(T_{k}T_{temp})}>\delta$, where δ takes a uniform distribution of [0, 1]. If the value of ΔF is smaller and the value of $T_{temp}$ is larger, the probability of the new solution being accepted is higher.

Step 5: Reduce system temperature $T_{temp}=\beta T_{temp}$, where β represents the temperature fading factor.

Step 6: Determine if the current temperature of the system $T_{temp}$ is less than the minimum temperature $T_{min}$. If $T_{temp}<T_{min}$, the algorithm terminates and outputs the optimal sensing waveform $S_{t}$. Otherwise, repeat steps 2 to 5 until $T_{temp}<T_{min}$.
**Algorithm 1** The simulated annealing algorithm. **Initialization:**     Generate a U randomly according to (10) to obtain $S_{t}$;     $T_{temp}=T_{max}$; **Repeat:**    Generate a new $U^{\prime }$ according to (9), U and $T_{temp}$ to    obtain $S_{t}^{\prime }$;    $\Delta F=F\left(U^{\prime }\right)-F\left(U\right)$;  **If** ΔF≤0 **Then** $U=U^{\prime }$, $S_{t}=S_{t}^{\prime }$;  **Else** Accept $U^{\prime }$ and $S_{t}^{\prime }$ with a probability $e^{-\Delta F/(T_{k}T_{temp})}$;     $T_{temp}=\beta T_{temp}$; **Until** $T_{temp}<T_{min}$; **Output:** $S_{t}$;

After the above algorithm optimization, the designed sensing waveform is $S_{t}$. Considering a point target, the received signal $y_{l}\left(t\right)$ of the *l*th sensing symbol of the sensing waveform can be described as

(12) $$\begin{array}{cc} y_{l}\left(t\right)= & \sigma s_{n_{l}}^{\prime }\left(t-\tau \right)e^{j2\pi f_{0}\left(t-\tau \right)}e^{j2\pi f_{d_{t}}n_{l}T_{s}}+c_{l}\left(t\right)+n_{l}\left(t\right)\\ = & \sum_{m=0}^{N_{c}-1}\sigma e^{j2\pi f_{0}\left(t-\tau \right)}e^{j2\pi m\Delta f(t-\tau -n_{l}T_{s})}\\ & e^{j2\pi f_{d_{t}}n_{l}T_{s}}\mathrm{rect}\left[\frac{t-\tau -n_{l}T_{s}}{T_{s}}\right]+c_{l}\left(t\right)+n_{l}\left(t\right), \end{array}$$

where τ is the round-trip propagation time of the target. When subcarrier separation is performed, τ is required to be smaller than $T_{cp}$. $f_{d_{t}}$ is the Doppler frequency shift, $c_{l}\left(t\right)$ denotes the clutter of the *l*th sensing symbol, $n_{l}\left(t\right)$ is the thermal noise of the *l*th sensing symbol, and σ is the attenuation factor caused by propagation loss, scattering, and the radar cross-section of the target.

After performing the operations of carrier frequency removal, sampling, and subcarrier separation [18] on $y_{l}\left(t\right)$, the resulting signal of *l*th sensing symbol is expressed as

(13) $$\begin{array}{cc} {\hat{y}}_{l}\left(m\right)= & N_{c}\sigma e^{j2\pi f_{d_{t}}n_{l}T_{s}}e^{-j2\pi f_{0}\tau }e^{-j2\pi m\Delta f\tau }+{\hat{c}}_{l}\left(m\right)+{\hat{n}}_{l}\left(m\right), \end{array}$$

where $m=0,\;1,\;\ldots ,\;N_{c}-1$. Therefore, the received sensing signal can be expressed in terms of a matrix by

(14) $$Y=a\omega_{f_{d}}\omega_{\tau }+C+N$$

where $y_{m}={\left[{\hat{y}}_{0}\left(m\right),\;{\hat{y}}_{1}\left(m\right),\;\ldots ,\;{\hat{y}}_{L-1}\left(m\right)\right]}^{T}$, $Y=[y_{0},\;y_{1},\;\ldots$, $y_{N_{c}-1}]$, $\omega_{f_{d}}=[e^{j2\pi f_{d}n_{0}T_{s}},\;e^{j2\pi f_{d}n_{1}T_{s}}$, $\ldots ,\;e^{j2\pi f_{d}n_{L-1}T_{s}}{]}^{T}$, $a=N_{c}\sigma e^{-j2\pi f_{0}\tau }$, $\omega_{\tau }=\left[1,\;e^{-j2\pi 1\Delta f\tau },\;\ldots ,\;e^{-j2\pi \left(N_{c}-1\right)\Delta f\tau }\right]$, $c_{l}=[{\hat{c}}_{l}\left(0\right),\;{\hat{c}}_{l}\left(1\right)$, $\ldots ,\;{\hat{c}}_{l}\left(N_{c}-1\right){]}^{T}$, $C={\left[c_{0},\;c_{1},\;\ldots ,\;c_{L-1}\right]}^{T}$, $n_{l}={\left[{\hat{n}}_{l}\left(0\right),\;{\hat{n}}_{l}\left(1\right),\;\ldots ,\;{\hat{n}}_{l}\left(N_{c}-1\right)\right]}^{T}$, $N=[n_{0},\;n_{1},\;\ldots$, $n_{L-1}{]}^{T}$. In the next section, we will perform the detection and estimation of the received sensing signal based on Y in (14).

### 3.3. Signal Detection and Estimation

**Figure 4:**
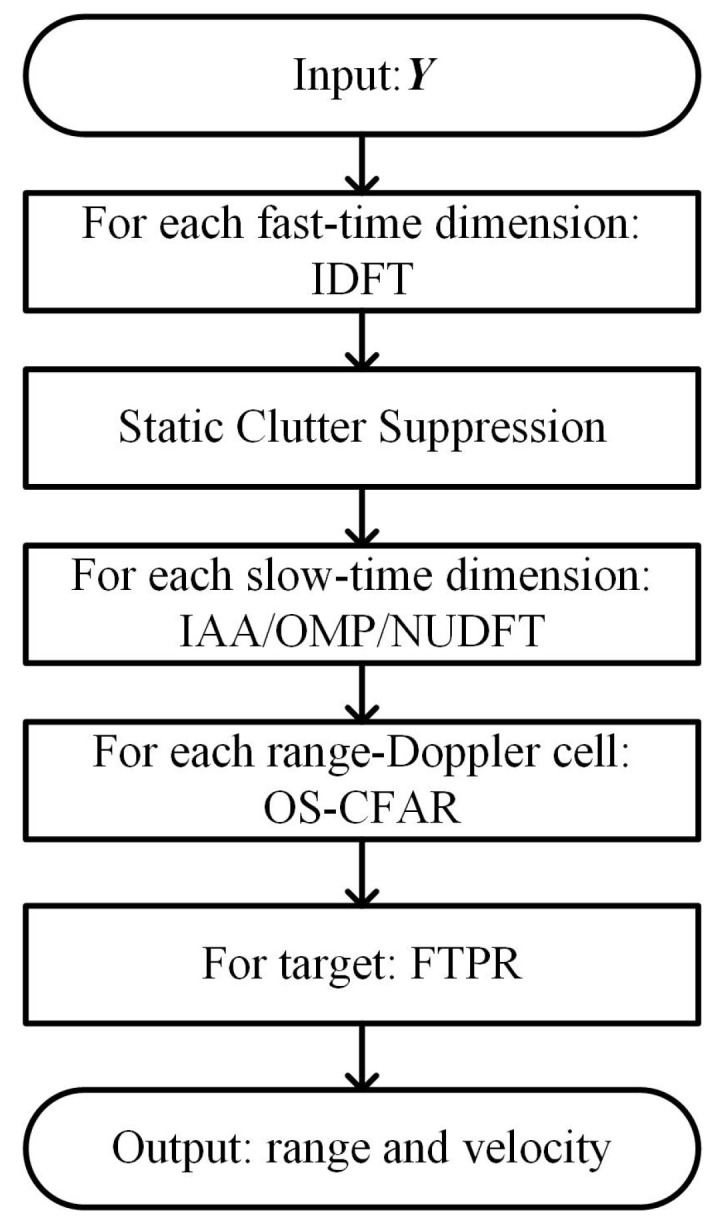
Signal processing flowchart.

In this section, we detail the sensing signal detection and estimation using the designed waveform. The specific workflow is illustrated in Figure 4. After preprocessing the received signal to obtain Y as shown in (14), we perform the corresponding signal processing on Y to achieve target detection and parameter estimation. First, we perform an inverse discrete Fourier transform (IDFT) on the fast-time dimension of Y to obtain range data. We then apply static clutter suppression on the slow-time dimension. Sparse recovery or a non-uniform discrete Fourier transform (NUDFT) is performed on the slow-time dimension to obtain Doppler data. Subsequently, the ordered-statistic constant false-alarm rate (OS-CFAR) is applied to each range-Doppler cell. Once a target is detected, frequency-time phase regression (FTPR) is conducted to obtain the target’s range and velocity. Specific details are as follows.

We calculate the range spectrum by IDFT along the fast-time dimension of Y.

(15) $$\overset{ˇ}{Y}=YW,$$

where W is the inverse discrete Fourier transform matrix, given by

(16) $$W=\left[\begin{array}{cccc} 1 & 1 & \ldots & 1\\ 1 & e^{j2\pi 1/N_{c}} & \ldots & e^{j2\pi \left(N_{c}-1\right)/N_{c}}\\ \vdots & \vdots & \ddots & \vdots \\ 1 & e^{j2\pi \left(N_{c}-1\right)/N_{c}} & \ldots & e^{j2\pi \left(N_{c}-1\right)\left(N_{c}-1\right)/N_{c}} \end{array}\right]$$

Let $\overset{ˇ}{Y}=\left[{\overset{ˇ}{y}}_{0},\;{\overset{ˇ}{y}}_{1},\;\ldots ,\;{\overset{ˇ}{y}}_{N_{c}-1}\right]$, where ${\overset{ˇ}{y}}_{m}$ is the received data of the *m*th range cell. We then perform the static clutter suppression on ${\overset{ˇ}{y}}_{m}$ along the slow-time dimension, as given by

(17) $${\hat{y}}_{m}={\overset{ˇ}{y}}_{m}-{\bar{y}}_{m}v_{L},$$

where ${\bar{y}}_{m}=1/L\sum_{l=0}^{L-1}{\overset{ˇ}{y}}_{m}\left(l\right)$, and $v_{L}$ is the L×1 vector, with all elements equal to one. It is clear that ${\hat{y}}_{m}$ is sparsely sampled in the Doppler dimension. According to compressed sensing (CS) theory, if a signal is sparse or compressible in a certain transform domain, it may be reconstructed using nonlinear methods by solving an optimization problem with high probability. Furthermore, the problem can be abstracted as recovering the vector ${\tilde{y}}_{m}$ from data ${\hat{y}}_{m}=A{\tilde{y}}_{m}$, where A is required to satisfy the restricted isometry property (RIP) [34]. Specifically, $A=[a\left(f_{1}\right),\;a\left(f_{2}\right),\;\ldots ,\;a\left(f_{K}\right)]$ is the temporal steering vector dictionary, where $A\left(f_{k}\right)={\left[e^{j2\pi f_{k}n_{0}T_{s}},\;\ldots ,\;e^{j2\pi f_{k}n_{L-1}T_{s}}\right]}^{T}$ is the steering vector. From [35,36], it is known that A satisfies RIP with high probability if the eigenvalues of the Gram matrix $A_{\Gamma }^{H}A_{\Gamma }$ are bounded near 1 for every Γ (Γ is a subset of {1…K}). Since it is difficult to prove the temporal steering vector dictionary A satisfying the RIP, we compare the eigenvalues of its Gram matrices to that of a matrix with Gaussian entries of zero mean and variance 1/L after scaling A, so that its columns have unit norm [34]. According to the CS theory, the Gaussian random matrix satisfies the RIP with high probability [37].

**Figure 5:**
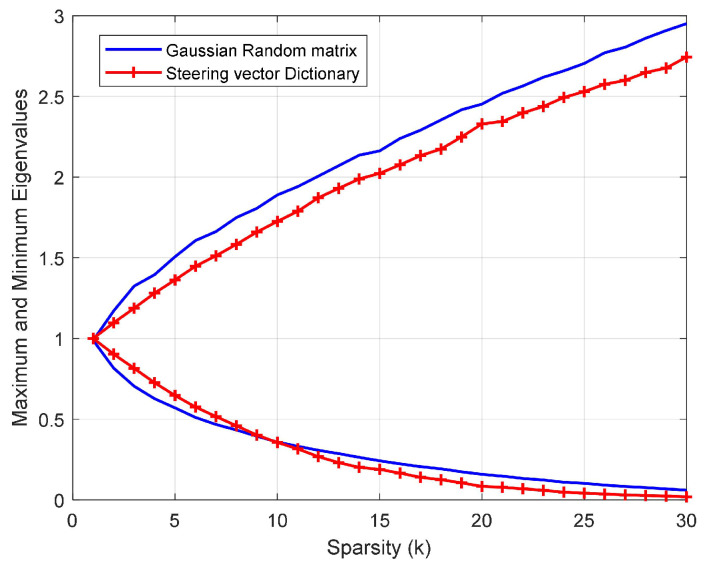
Maximum and minimum eigenvalues of the Gram matrix against sparsity k.

As shown in Figure 5, the maximum and minimum eigenvalues of the Gram matrix $A_{\Gamma }^{H}A_{\Gamma }$ are close to 1, and are similar to the eigenvalues of the Gaussian random matrix. Therefore, A satisfies the RIP with high probability, and ${\tilde{y}}_{m}$ can be reconstructed with high probability in the dictionary A.

Here, we introduce two sparse recovery algorithms, namely the iterative adaptive approach (IAA) and the orthogonal matching pursuit (OMP) to reconstruct ${\tilde{y}}_{m}$.

As for the IAA algorithm, it transforms the solving of the Doppler frequency of the target in ${\hat{y}}_{m}$ into solving the following optimization problem:

(18) $$\min\;||{\hat{y}}_{m}-\gamma_{k}a\left(f_{k}\right){\left|\right|}_{Q_{k}^{-1}}^{2}$$

where ${\left||x|\right|}_{Q_{k}^{-1}}^{2}=x^{H}Q_{k}^{-1}x$, $Q_{k}$ denotes the covariance matrix of the clutter and noise, and $|\gamma_{k}{|}^{2}$ is the power of the Doppler spectrum with frequency $f_{k}$.

The IAA algorithm operates in an iterative way and the power of the Doppler frequency points $P_{k,k}$ is calculated for each range of interest in each iteration, and then the IAA covariance matrix $R_{\mathrm{IAA}}$ is updated. The derivation details can be seen in reference [38]. A summary of the IAA algorithm is shown in Algorithm 2.

As for the OMP algorithm, it operates in an iterative way and computes the maximum inner product via the matrix–vector multiplication $Ar_{p}$ for each iteration, where $r_{p}$ is the residual vector at the *p*th iteration. Moreover, the solution estimated at each iteration requires the solution of a least-squares problem, given by

(19) $$\min{∥A_{\Omega (p+1)}y_{\Omega (p+1)}-{\hat{y}}_{m}∥}_{2}^{2}$$

where $\Omega \left(p+1\right)=\{b\left(p_{i}+1\right)|p_{i}=1,\;2,\;\ldots ,\;p\}$ denotes the set consisting of b(p+1) for each iteration. The derivation details can be seen in reference [39]. A summary of the OMP algorithm is shown in Algorithm 3.
**Algorithm 2** The IAA algorithm. **Initialization:**    $A=[a\left(f_{1}\right),\;\ldots ,\;a\left(f_{K}\right)]$,    IAA covariance matrix $R_{\mathrm{IAA}}=I$,    $P_{k,k}=\frac{1}{L^{2}}{\left|a^{H}\left(f_{k}\right){\hat{y}}_{m}\right|}^{2}$, k=1, … ,K. **Repeat:**    $R_{\mathrm{IAA}}=\mathrm{AP}A^{H}$,    **For** k=1, …, K,         $P_{k,k}=|\gamma_{k}{|}^{2}={\left|\frac{a^{H}\left(f_{k}\right)R_{\mathrm{IAA}}^{-1}{\hat{y}}_{m}}{a^{H}\left(f_{k}\right)R_{\mathrm{IAA}}^{-1}a\left(f_{k}\right)}\right|}^{2}$,    **End** **Until** a certain number of iterations is reached. **Output: **${\tilde{y}}_{m}=diag\left(P\right)$.

**Algorithm 3** The OMP algorithm.
  **Initialization:**
     ${\hat{y}}_{m}$, $A=[a\left(f_{1}\right),\;\ldots ,\;a\left(f_{K}\right)]$, p=0,
     Residual vector $r_{0}={\hat{y}}_{m}$,
     Stopping parameter ϵ,
     Selected index subset Ω(0)=∅.
 **While** $\left|\right|r_{p}{\left|\right|}_{2}^{2}>\epsilon$
     $b\left(p+1\right)={\mathrm{argmax}}_{b}\left|a^{H}\left(f_{b}\right)r\left(p\right)\right|$,
     Ω(p+1)=Ω(p)∪b(p+1),
     $G={\left(A_{\Omega (p+1)}^{H}A_{\Omega (p+1)}\right)}^{-1}A_{\Omega (p+1)}^{H}$,
     $y_{\Omega (p+1)}=G{\hat{y}}_{m}$,
     Update the residual vector $r_{p+1}={\hat{y}}_{m}-A_{\Omega (p+1)}y_{\Omega (p+1)}$,
     p=p+1,
 **End**
Compute the Doppler profile ${\tilde{y}}_{m}=B_{s}y_{\Omega (p+1)}$.


After applying the sparse recovery algorithm to ${\hat{y}}_{m}$, we obtained the Doppler dimension data ${\tilde{y}}_{m}$ for the *m*th range cell. Then, the location of the target is detected on the range-Doppler map by the OS-CFAR [40] detector. The specific process of OS-CFAR is as follows: First, for the detection cell $z_{m,k}$ in the range-Doppler map, we select some cells around the detection cell as the protection cell. We then select some cells that are not in the scope of the protection cells as reference cells. Moreover, we sort the reference cells according to their power, and then select the middle $N_{\mathrm{CFAR}}$ cells of the reference cells to estimate the power of clutter and noise $P_{\mathrm{CN}}$. Subsequently, we obtain the detection threshold $T_{\mathrm{CFAR}}$ by multiplying $P_{\mathrm{CN}}$ by a factor ζ. If the power of $z_{m,k}$ is greater than $T_{\mathrm{CFAR}}$, it means that the target exists, otherwise, it means that the target does not exist.

Finally, we propose using the FTPR algorithm [41] to estimate the distance and velocity of the target. Specifically, we assume that the target is detected at the $m_{t}$th distance cell and the $k_{t}$th Doppler cell. We then obtain the $\phi_{m_{t}}^{\prime }$ by taking the Doppler spectrum $\phi_{m_{t}}$ of the $m_{t}$th range cell and setting the values of Doppler cells outside $\left[k_{t}-1,\;k_{t}+1\right]$ to zero, as given by

(20) $$\phi_{m_{t}}^{\prime }=\left\{\begin{array}{c} \phi_{m_{t}},\;k\in \left[k_{t}-1,\;k_{t}+1\right]\\ 0,\;k\notin [k_{t}-1,\;k_{t}+1] \end{array}\right.$$

An inverse fast Fourier transform (IFFT) is performed on $\phi_{m_{t}}^{\prime }$ and the Doppler frequency $f_{d_{t}}$ is obtained by finding the phase slope, given by

(21) $$f_{d_{t}}=\frac{\mathrm{Slope}(∠\mathrm{IFFT}\left[\phi_{m_{t}}^{\prime }\right])}{2\pi },$$

where IFFT[x] denotes the IFFT of *x*, ∠x stands for the phase of *x*, and Slope(x) represents the slope of *x*. The speed of the target $\hat{v_{t}}$ is estimated by

(22) $$\hat{v_{t}}=\frac{f_{d_{t}}\lambda }{2},$$

where λ is the wavelength. Similarly, the distance of the target $\hat{r_{t}}$ is as follows:

(23) $$\hat{r_{t}}=\frac{f_{r_{t}}c}{2\Delta fB},$$

where *c* is the propagation velocity, *B* is the bandwidth.

Regarding complexity, this paper focuses on analyzing the computational complexity of the NUDFT, DFT, IAA, and OMP. Since the number of iterations is difficult to predict, we consider the complexity per iteration, and study the convergence of the algorithms in terms of iterations, numerically, in our results. In all the computations, we only consider the highest-order terms and omit the low-order ones. For the NUDFT, there exists a fast algorithm NUFFT, which reduces the complexity of the method from O(LK) to O(Llog(K)). Similarly, DFT also has a fast Fourier transform (FFT) with a computational complexity of O(Llog(L)). For IAA, its complexity is $O(L^{3}K^{3})$. Reference [38] suggests that after more than 10 iterations of IAA, its performance does not significantly improve. For OMP, its complexity is $O(L^{2}K)$. The number of iterations of OMP is affected by the sparsity of the target, and in the simulation experiments of this paper, the number of iterations is generally no more than 10 times.

## 4. Numerical Examples

**Figure 6:**
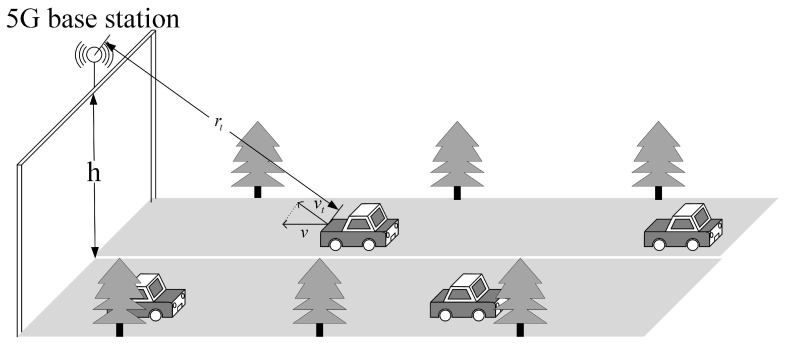
Simulation of traffic scenarios.

In this section, several numerical examples are given to verify the performance of the designed waveform. We demonstrate the performance of the designed waveforms through three aspects, namely waveform parameter impacts, detection performance, and estimation performance. In the following experiments, we consider a 5G BS mounted on a gantry (with height denoted as *h*) to detect and estimate the parameters of a vehicle that is underneath. The simulation of traffic scenarios is shown in Figure 6, where *v* denotes the forward velocity of the vehicle, $v_{t}$ denotes the radial velocity of the vehicle, and $r_{t}$ denotes the radial range of the vehicle. Therefore, the clutter is modeled as a Gaussian clutter model [42], and the thermal noise is complex Gaussian noise with zero mean. The mounting height of 5G BS is h=7 m, the wind velocity in the clutter model is $v_{w}=2$ m/s, the 5G system parameter is μ=3, the subcarrier interval is Δf=120 KHz, the bandwidth is B=400 MHz, and the carrier frequency is $f_{0}=26$ GHz. Therefore, T=8.33 μs, $T_{cp}=0.58\;\mu$s, and the maximum range of detection is $R_{max}=T_{cp}c/2=87.5$ m.

### 4.1. Waveform Parameters Impacts

**Figure 7:**
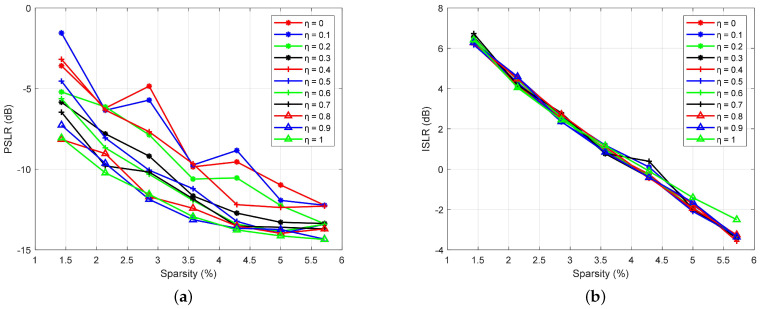
Sidelobe intensity vs. sparsity. (**a**) PSLR vs. sparsity for different η factors. (**b**) ISLR vs. sparsity for different η factors.

First, we evaluate the PSLR and the ISLR of the velocity ambiguity functions of the designed waveforms in terms of different sparsity $K_{s}$ and scaling η factors. The results are shown in Figure 7. It can be seen from the figure that the PSLR and the ISLR of the velocity ambiguity function of the designed waveform gradually decrease as the sparsity increases, which illustrates that the sidelobe leakage decreases as the number of OFDM symbols increases.

In Figure 7a, we can see that the PSLR decreases as η increases. Moreover, when $K_{s}\geq 4.28\%$ and η≥0.5, the PSLR remains relatively stable. In addition, when η≤0.2, the PSLR appears to fluctuate as $K_{s}$ increases. The reason for this is that the weight of PSLR in the cost function is too small, which makes the PSLR not well-optimized. In Figure 7b, the ISLR of the velocity ambiguity function of the designed waveform does not fluctuate significantly as η increases, except for η=1. The reason for this is that the optimization of the ISLR is not considered in the optimization algorithm when η=1.

**Figure 8:**
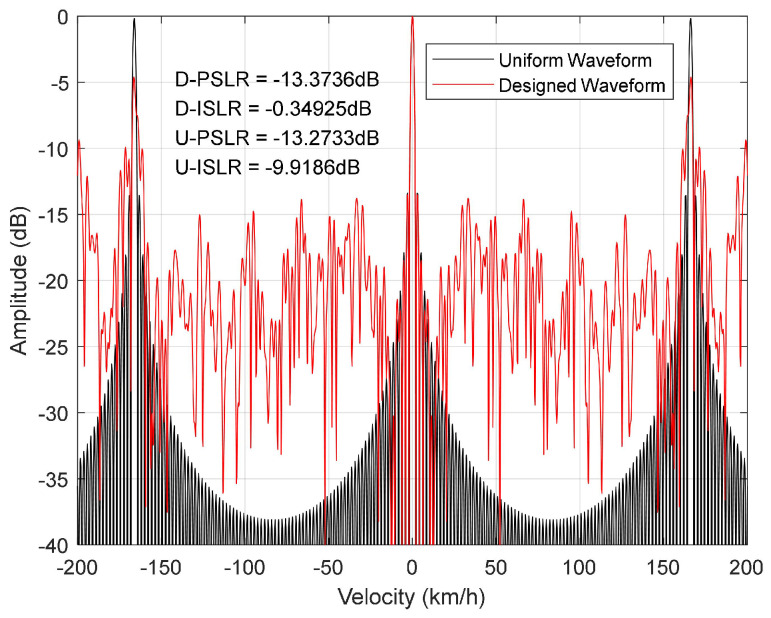
Velocity ambiguity function.

Next, we compare the performance of the velocity ambiguity function for the designed waveform between $K_{s}=4.28\%$, η=0.6 and that of the uniform waveform, which has a total of 80 OFDM symbols, each equally spaced within a wireless frame. The timings of the designed waveform and the uniform waveform are shown in Table 1. The row where the designed waveform and the uniform waveform are located indicates that the *i*th OFDM symbol within a slot is extracted as the sensing symbol. There are 80 slots within a wireless frame when the 5G system parameter μ=3. And ’null’ indicates that there is no sensing symbol in the corresponding slot. We note that the uniform waveform does not satisfy the constraint in (9). The velocity ambiguity functions are shown in Figure 8. It can be seen from the figure that the velocity resolution is 2.08 km/h for both the designed waveform and uniform waveform, and the maximum unambiguous velocity is ±83.09 km/h. Moreover, the PSLR of the designed waveform’s velocity ambiguity function is −13.37 dB, which is close to the PSLR of the uniform waveform. However, it should be noted that the ISLR is −0.35 dB, which is much higher than the ISLR of −9.92 dB for the uniform waveform. This means that the designed waveform has a sidelobe leakage problem, but the peaks of the sidelobe are all low.

In the next experiment, we analyze the sparse recovery performance of the designed waveform for different target numbers. We set SCNR = −10 dB, the number of Doppler cells is set to 320, and the target numbers are set to $N_{t}$ = 4, 8, and 12, respectively. And the signal-to-clutter-plus-noise ratio (SCNR) is expressed by (24), according to (14).

(24) $$\mathrm{SCNR}=10lg\frac{tr[{\left(a\omega_{f_{d}}\omega_{\tau }\right)}^{H}a\omega_{f_{d}}\omega_{\tau }]}{tr\left(C^{H}C\right)+tr\left(N^{H}N\right)}$$

**Figure 9:**
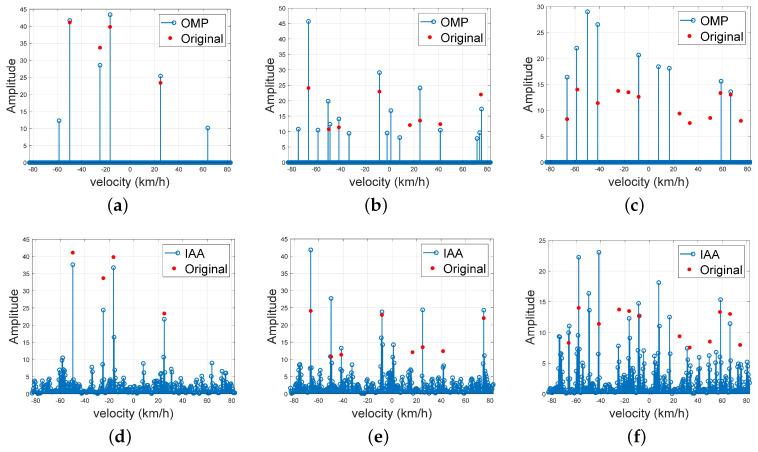
Velocity dimension with OMP and IAA for targets of different numbers. (**a**,**d**) $N_{t}$ = 4. (**b**,**e**) $N_{t}$ = 8. (**c**,**f**) $N_{t}$ = 12.

The results are shown in Figure 9. Figure 9a and Figure 9d represent the velocity dimension for $N_{t}$ = 4 targets and the results for $N_{t}$ = 8 and $N_{t}$ = 12 targets are shown in Figure 9b, Figure 9e, Figure 9c, and Figure 9f, respectively. It can be seen from the figure that when the number of targets is not too large, the OMP and IAA methods can recover the velocity of the targets accurately, with a good suppression effect on the sidelobe. As the number of targets increases, the sparse recovery performance decreases. When the number of targets increases to 12, a small number of targets cannot be well recovered, and some spurious targets also appear. This is because the targets no longer satisfy the sparse characteristics for the designed waveform with only 48 OFDM symbols.

### 4.2. Detection Performance

Here, we illustrate the detection performance of the designed waveform and uniform waveform. We apply NUDFT, IAA, and OMP to verify the detection performance of the designed waveform, and the sparsity $K_{s}=4.28\%$ among these algorithms. We apply the DFT, IAA, and OMP to verify the detection performance of the uniform waveform. In order to achieve the threshold of false alarm probability (Pfa), 30,000 Monte Carlo experiments are performed. Moreover, 500 independent Monte Carlo experiments are performed per SCNR in order to achieve the detection probability (Pd). In each Monte Carlo experiment, the radial velocity of the target is $v_{t}=45$ km/h, the radial range is $r_{t}=30$ m, the CNR is 30 dB, and the Pfa is 0.001.

**Figure 10:**
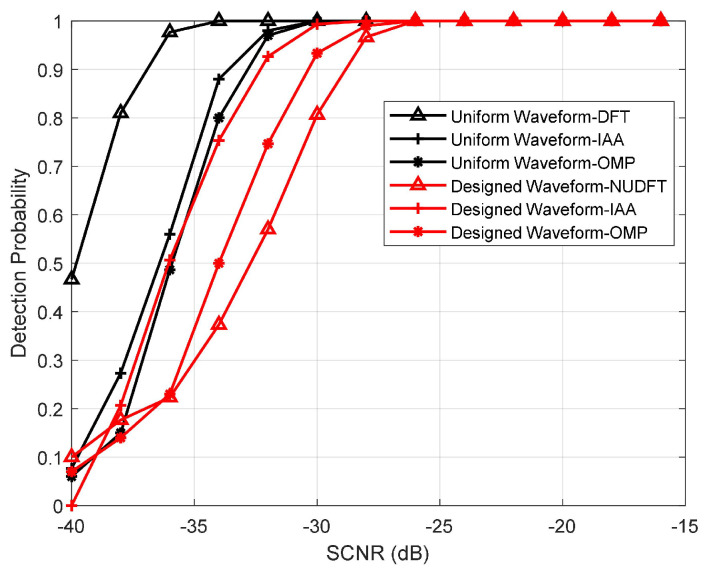
Pd vs. SCNR for different waveforms and algorithms.

The results are shown in Figure 10. The figure illustrates that the Pd of the designed waveform is over 95% when SCNR > −25 dB and Pfa = 0.001. It should be noted that compared to the IAA, the OMP and NUDFT have a 3 dB loss in the detection performance of the designed waveform when Pd =90%. And the results also illustrate that the detection performance of the uniform waveform with DFT is optimal when SCNR < −25 dB. However, we note that the Pd of the designed waveform is lower than that of the uniform waveform when the SCNR < −25 dB. Specifically, for the IAA, the detection performance of the designed waveform has a performance loss of less than 1 dB compared to the uniform waveform. For the OMP and NUDFT, the detection performance loss reaches more than 3 dB, compared to the OMP and DFT with uniform waveform. This is due to the non-sparsity of the clutter, and the noise distribution has an impact on the reconstruction of the target information in the case of a low SCNR ratio, which leads to the decrease of Pd. Moreover, the decrease in the number of sensing symbols similarly decreases Pd.

**Figure 11:**
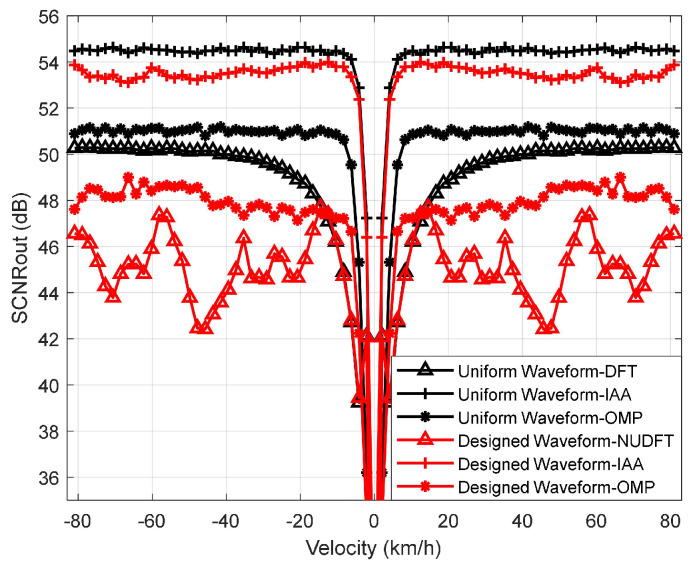
SCNRout vs. velocity for different waveforms and algorithms.

In the next experiment, we evaluate the detection performance of the designed waveform and the uniform waveform in the case of a high SCNR. The SCNR = 0 dB, and the other parameters are the same as in the last experiment. Since the Pd converges to 100% for SCNR > −25 dB, we define the output of SCNR (SCNRout) to describe the intensity contrast relationship between the signal, clutter, and noise after the signal processing. The SCNRout can be defined as

(25) $$\mathrm{SCNRout}=10lg\frac{P_{t}}{mean(P_{ref})}$$

where $P_{t}$ is the power of the target, and $P_{ref}$ is the power of the reference cell. The result is shown in Figure 11. The figure illustrates that the detection performance of the designed waveform using the IAA is better than that of applying the OMP and NUDFT, which is similar to the results of the last experiment. Different from the last experiment, the detection performance of the uniform waveform applying IAA is the best. Moreover, the detection performance of the designed waveform and the uniform waveform applying IAA is close. This is because, as the SCNR increases, the non-sparsity of the clutter and noise distribution has less influence on the target information. The information of the target is more easily brought out, making the sparsity of the signal more obvious. Similar to the conclusion in Figure 5, the sparser the signal is, the higher the probability that the signal can be reconstructed by the sparse recovery algorithm. And the IAA employs sparse prior knowledge so that the IAA is more robust to the reduction in the number of sensing symbols compared to the OMP, DFT, or NUDFT. In addition, we note that the SCNRout curve of the designed waveform applying NUDFT is not smooth. The reason for this is the non-uniformity of the designed waveform. According to the second and third experiments, we can see that the detection performance applying OMP decreases as the waveform sparsity decreases.

### 4.3. Estimation Performance

**Figure 12:**
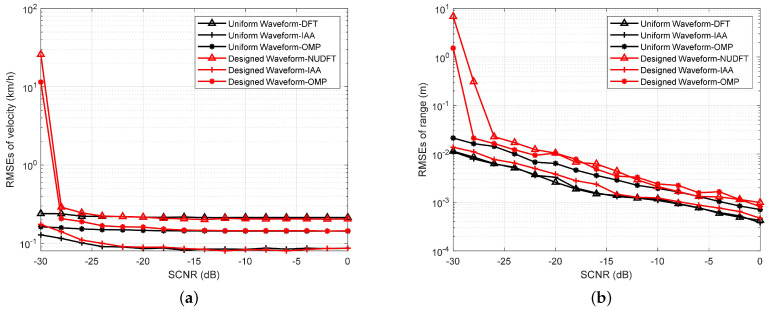
RMSEs vs. SCNR. (**a**) RMSEs of velocity estimations vs. SCNR for different waveforms and algorithms. (**b**) RMSEs of range estimations vs. SCNR for different waveforms and algorithms.

In this experiment, we discuss the estimation accuracy of the designed waveform. In order to achieve the root mean square errors (RMSEs) of range and velocity estimations, 500 independent Monte Carlo experiments are performed per SCNR. The RMSEs of the range can be defined as follows:

(26) $$R_{rmse}=\sqrt{\frac{1}{N_{e}}\sum_{N_{e}}{\left|\hat{r_{t}}-r_{t}\right|}^{2}},$$

where $N_{e}$ is the number of Monte Carlo experiments, $\hat{r_{t}}$ is the estimated value of the range, and $r_{t}$ is the actual value of the range. Similarly, we can obtain the RMSEs of the velocity by replacing the estimated and actual values of the velocity into (26). In each Monte Carlo experiment, the velocity of the target is generated randomly. The other parameters are the same as the detection performance experiment. The results are shown in Figure 12. The plots show that the RMSEs of velocity and range estimations decrease with an increase in the SCNR. Moreover, the designed waveform and the uniform waveform applying IAA have very close velocity and range estimation accuracies.

In Figure 12a, we notice that the RMSEs of the velocity of the designed waveform using the same algorithm are slightly worse than the RMSEs of the velocity of the uniform waveform when SCNR < −22 dB. The reason for this is that the ISLR of the velocity ambiguity function of the uniform waveform is smaller, and the accuracy is higher when FTPR is adopted. When SCNR >−22 dB, the RMSEs of the velocity of the designed waveform and the uniform waveform are similar when applying the same algorithm. In addition, the RMSEs of the velocity of the IAA method are better than that of the OMP method, which are better than that of the DFT or NUDFT methods. The reason for this is that the IAA and OMP methods are able to suppress the sidelobe.

In Figure 12b, we notice that the RMSEs of range decrease as the SCNR increases. The RMSEs of range for the designed waveform and the uniform waveform applying the IAA are very similar to the results of the RMSEs of velocity. However, the difference is that the RMSEs of the range of the designed waveform applying the OMP and NUDFT are slightly worse than those of the uniform waveform applying the OMP and DFT. This is due to the reduction in the number of sensing symbols, which reduces the performance of the range estimation, applying the OMP and NUDFT methods.

## 5. Conclusions

In this paper, we propose an inner-frame time division multiplexing waveform design method of ISAC in the 5G NR system, which achieves ISAC by reusing the hardware of existing 5G BSs. The proposed waveform design strategy describes the inner-frame time division multiplexing waveform design as an optimization problem of a constrained combination of PSLR and ISLR. Specifically, the constraints are the 5G communication protocol and 5G NR frame structure. This optimization problem is solved by the simulated annealing algorithm, and the corresponding signal detection and estimation methods based on sparse recovery techniques are developed to obtain the target information. The experiments show that the designed waveform satisfies the downlink and common channel constraints and has a 40% reduction in sensing costs compared to the uniform waveform. In terms of theoretical performance, the PSLR of the velocity ambiguity function, the velocity resolution, and the ambiguity-free velocity range are similar to those of the uniform waveform. Additionally, the designed waveform using the IAA can achieve a detection and estimation performance close to that of the uniform waveform in the case of high SCNR.

## Figures and Tables

**Table 1:** Designed Waveform Timing.

Slot	0	1	2	3	4	5	6	7
Designed waveform	3	0	null	13	null	null	13	13
Uniform waveform	0	0	0	0	0	0	0	0
Slot	8	9	10	11	12	13	14	15
Designed waveform	12	null	null	0	13	11	null	null
Uniform waveform	0	0	0	0	0	0	0	0
Slot	16	17	18	19	20	21	22	23
Designed waveform	3	1	0	null	null	1	0	0
Uniform waveform	0	0	0	0	0	0	0	0
Slot	24	25	26	27	28	29	30	31
Designed waveform	null	1	null	13	12	null	0	null
Uniform waveform	0	0	0	0	0	0	0	0
Slot	32	33	34	35	36	37	38	39
Designed waveform	13	13	null	0	null	1	0	null
Uniform waveform	0	0	0	0	0	0	0	0
Slot	40	41	42	43	44	45	46	47
Designed waveform	1	0	null	0	null	null	3	1
Uniform waveform	0	0	0	0	0	0	0	0
Slot	48	49	50	51	52	53	54	55
Designed waveform	0	null	null	5	3	3	null	null
Uniform waveform	0	0	0	0	0	0	0	0
Slot	56	57	58	59	60	61	62	63
Designed waveform	5	4	3	null	3	2	null	0
Uniform waveform	0	0	0	0	0	0	0	0
Slot	64	65	66	67	68	69	70	71
Designed waveform	null	null	11	12	10	null	12	11
Uniform waveform	0	0	0	0	0	0	0	0
Slot	72	73	74	75	76	77	78	79
Designed waveform	null	11	null	11	null	13	10	null
Uniform waveform	0	0	0	0	0	0	0	0

## Data Availability

Not applicable.
